# Lecture on “Calcarea”

**Published:** 1885-08

**Authors:** J. T. Kent

**Affiliations:** St. Louis


### LECTURE ON "CALCAREA."

PROFESSOR J. T. KENT, M. D., ST. LOUIS.

CHARACTERISTICS.—Leucophlegmatic constitution; imperfect formation of bone. Indigestion, caused by lime-water, will be cured by lime in dynamic form. (In Sulphur the symptoms are largely physical; in Aconite largely mental.) In Calcarea they are both physical and mental. The leading mental symptom is this: "She fears she will lose her reason, or that people will observe her mental confusion." She reasons clearly, but thinks others observe mental aberration in her. Shuddering and dread as the evening draws near. The mental state is aggravated *in the evening*. Great anxiety and palpitation of the heart. Gloomy and *melancholy*. Forgetful; misplaces words. Talks about mice, rats, and murder. Disinclination for work. She will continue brooding day and night. Evil forebodings. This mental state is not in every picture of Calcarea, but if there are mental symptoms they will be of this type. She takes cold easily, suffers from every change of the weather and from every draught of air. This mental state is likely to occur in old dyspeptics; in weakness of the brain tissue brought on by excessive mental application. The vertigo and mental symptoms are made *worse* by *close application of the mind, and in the evening*. Calcarea, as well as Sulphur, must become a very useful remedy in softening of the brain.

HEADACHE.—Beginning in the occiput and spreading to the vertex; feels as if she would go crazy. The pain in the head brings on this insane feeling. Throbbing headache in the middle of the brain. Many of these head symptoms are brought on by catarrhal states. The chronic catarrh is *moist*, but every cold makes it *dry*. Dryness and burning in the nose. Whenever the flow is stopped on come the head pains. Burning and splitting soreness in side of head, feeling as if the head would burst. Worse on *going up-stairs, talking*, or *walking; better* from *tight bandaging*. (This is similar to Arg-nit.) Headache *better after vomiting* (Sang.), *lying down*, or *pressure* of *the cold hand*. Icy coldness on the top of the head, most frequently on the *right side*. (Veratrum has a sensation of a lump of ice on the vertex.) *Headache coming on from suppressed nasal catarrh. Gets relief from vomiting.* Whitish-yellow scales of dandruff. *Head feels cold. Large head and open fontanelles.* Little infants, with late-closing of fontanelles, sometimes reopening after closing (Calc. or Silicea). Bell is the remedy for large-headed subjects in acute states; Calc. to prevent the recurrence. Burning on top of the head (Sulph). Calc. has sometimes hot feet, but it is the exception. Coldness of the head prominent, heat and burning would be the exception. There is a heat of the scalp, but it is superficial. (Sulph. patient feels as if she would like to put a cold cloth on her head.)

EYE.—Photophobia; Bell. for the acute, Calc. for the chronic. Entrance of a foreign body into the eye (also, Aconite for the acute inflammatory state), but when there is opacity and thickening, white substance forming on the eye and ulceration, Calc. Fungus hæmatodes, maculæ, suppurating fistula lachrymalis. Calcarea is capable of curing all the catarrhal states of the eye when its general symptoms are present.

EAR.—Calc. has cured hardness of hearing and ringing in the ears produced by taking Quinine. This will be met by China; also, Quinine produces a partial closure of the Eustachian tubes. Patient gets dizzy on going up-stairs. Singing, roaring, and crackling in the ears; crackling in the ears when chewing. Purulent offensive discharge from the ears, sometimes thick yellow muco-pus. Polypus in the ears. In connection with these symptoms you will find earache. Every time patient lies on right side the pain goes to that side, and *vice versa*.

I seldom give any other remedy than Puls. for earache.

For sties—Calc. and Puls.; if sty is indurated—Staph. and Thuja.

NOSE.—Calc. has a very large sphere of action in catarrhal conditions. (Calc.-Sulph. has a bloody discharge.) The Calc. patient sneezes and takes cold easily. (In Nux vomica the nose is *dry* at *night.*) Calc. has this as a chronic state. Nasal polypus.

FACE.—Pale, blue rings around the eyes; face feels as if it were swollen.

Men who have heavy beards, with itching in beard (if itching is aggravated by washing—Sulphur). The discharges of Calc. are likely to be offensive.

*Chewing motion of lower jaw;* children with open fontanelles; you might expect cerebral dropsy. Hydrocephalus.

SLOW DENTITION.—If the teeth come early, the fontanelles will close late, and *vice versa.* Children late in teething and learning to walk (late learning to talk—Natrum-mur.).

The taste is *sour.* Calc. in a general way is a *sour* remedy. The stool is *sour,* the taste is *sour,* the whole body is *sour* (Hepar is generally given in that state), the urine even smells SOUR.

THROAT.—Chronic sore throat, with dryness, pains extending to the ears; with this there may be inflammatory swelling of the palate, with sensation of contraction in throat when swallowing. (Lach. and Apis. left side.) Gels. has a pain running to the ears. Hepar also, with a sensation of a fishbone in the throat.

He feels the crawling sensation of a worm in the rectum and a *desire for eggs.* (Desire for wine—Nux-vom. and many other remedies. Craving for salt things—Natrum-mur. and *Selen.*)

Loss of appetite, but when he begins to eat, eats heartily (Cham.). (Craves things which, when obtained, he rejects—Bry.).

Milk disagrees—forms a curd in the stomach. (Æthusa has a slimy, sleek-looking curd, like "schmir käse.") Nausea and vomiting. Eructations taste of the food and *sour.* (Ferrum—Eructations tasting of the food just eaten.) Burning from stomach to throat, with eructations; these are so *sour* that they cause this burning. (If the burning is continuous, extending to mouth and stomach—Iris.) Vomiting, mostly in the morning and toward evening, during dentition. Where there is vomiting in pregnancy, with *cold, damp feet, etc., worse* in the evening; may cure if it occurs in the morning. (Sulph. vomit is aggravated in the evening.) (If patient is made sick every time she puts anything warm in the stomach—Phos. and Puls.) (Pressing pain in the stomach as if a load or stone was there—Nux, Puls., and Bry. Nux has this feeling an hour after eating. Abies nigra has it immediately after eating. Graphite pain comes on as soon as the stomach is empty, and the *pain* drives her to eat.)

Pit of stomach swollen like an inverted saucer; stomach is hard. Feeling of coldness in the abdomen (Plumbum, Ars., Colchicum), mesenteric glands swollen and hard in children. Mental state is guiding when present. Sensation of cold, damp stockings.

STOOL.—Frequent; first hard, then pasty, then liquid; offensive, like putrid eggs; yellowish or clay-colored, whitish or watery; white, undigested, involuntary; hardly an effort is made toward retaining stool. (Podo. has a white stool, clay-colored, with prolapsus.) Discharge of blood from rectum (Sulph. and Lyc.). Feeling of heaviness and weight in lower part of rectum (Nux vom.).

SEXUAL ORGANS.—Calc. is a basic remedy in a great many female troubles. It is a very important remedy in almost any sort of genital weakness; old cases of sexual abuse; system broken down; heart troubles; cold, relaxed sexual organs. Sexual desire at three A. M.; cold penis; slow erection with premature ejaculation (Nux-v., Sulph. and Lyc. all varying somewhat). Nocturnal pollutions, which debilitate both body and mind. Excessive exhaustion after copulation. Cold seems to go through him (Sepia). Gonorrhœa; continuous gleety discharge; afterward stricture.

MENSES.—Too early, too profuse, and last too long (very similar to Nux-v. Calc. patient flows more, while Nux patient has constipation and frequent desire to defecate). Inter-menstrual discharge of blood. Menses coming back too soon from excitement. Suppression of the menses with full habit after working in water. Membranous dysmenorrhœa.

LUNGS, COUGH, ETC.—A state that precedes the formation of tubercle. With the characteristics of Calc. you will find dropsy. You may think the patient is getting fat, but if he is examined the tissues will feel doughy, puffed up. The face has a doughy, waxy appearance; coughs every time he goes into a cold room; chilliness excites cough. If patient likes music, the excitement of it produces cough; expectoration has a putrid odor. Tickling cough; sensation of a feather in the throat. (Cough begins as soon as head touches pillow—Drosera.) (Cough as soon as gets warm in bed—Puls., Nux-m., and Coccus-c.) (Cough excited by going into a cold room—Phos. and Rumex.) All Calc. symptoms are made worse by taking cold. Stitches in chest and sides of chest when moving and when lying on affected side. (Bry.—better lying on painful side.) In connection with lung troubles rattling in chest. (For dry cough in emaciated boys, think of Lyc.) (In millers' and stonecutters' ailments, think of

Calc. and Silicea.) Old suppurating cavities and abscesses may need Calc. Hard swelling of cervical glands; thick, strumous tumefaction of thyroid gland; swelling and incurvation of the vertebral column; rachitis and caries of the spinal column. For injury in lower portion of spine—first, Arnica; second, Rhus, and then comes Calc., and it will do all that medicine can do. Whenever there is a sprain around a joint give Arnica, Rhus, and Calc. internally—giving time for each remedy to act. This is routine, but these remedies correspond to the transformations taking place in the joint. Calc. is a great remedy for gouty accumulations in the joints. Sulph., Calc., and Lyc. follow each other well in these gouty conditions, rheumatic gout, with scanty urine and constipation (Alum). Rheumatism brought on from exposure; patient is influenced by every change in the weather (Calc.-ph.). Aggravated from wet and cold. Sometimes fingers feel as if dead. Pain as if from a sprain in right wrist. (If patient kicks covers off and wants to be cool in rheumatism—Apis and Puls.) Sciatic pains caused by working in water. Pain is aggravated from limb hanging down and ameliorated from elevation of limb (milk-leg). Swelling of the knees. (Sulph. and Ledum also act upon the knees.) White swelling of legs and feet, with sensation of coldness. Abscesses feel cold. Cramps in the calves at three A.M.; also in popliteal space when extending limb. (Sulph. has cramps in the calves and in the soles of the feet.) In many of these complaints, you will be obliged to begin with Sulph. and then follow with Calc. Sulph. and Nitric acid are followed well by Calc. Burning in the soles (Sulph.). Sweat makes feet sore (Silicea). Paralytic bruised pain in the small joints.

NERVES.—Calc. is very important in the treatment of the nervous system, brain, and spinal affections (Sulph., Calc., and Lyc. in the treatment of epilepsy). Twitching of muscles, trembling of the body, talking produces a feeling of weakness, which compels the patient to desist. While he may feel well, as soon as he exerts himself or becomes excited he is exhausted. (The exhaustion of Arsenicum is quite *real;* patient feels so tired he cannot move.) The Calc. patient feels as though he could run a long race, but after he gets started the exhaustion comes on. It does not precede the motion, like *Ars.* Great exhaustion in the morning; unable to go up-stairs. Patient starts well enough, but before he gets half way up there comes on a difficulty of breathing. Going up a height produces great weakness. In epilepsy, before the attack, sensation of something running in the arm or from the pit of the stomach to the feet; feels like a mouse or large insect crawling in the skin.

In chills and fever, chill begins in pit of stomach and spreads over the body. Inclination to stretch in the morning.

Sleepiness and sleeplessness; awful dreams, anxious, fretful, dreams of falling. Children dream after midnight, cannot be pacified; cannot sleep after three A. M.; or late falling asleep, not until two or three A. M. (Sulph., Nux-v., and Kali carb. have a three A. M. aggravation.) Sensation of trembling in the inner parts on awakening from sleep. (Bell. is *worse* from sleep; there is a turgid state in the entire system; tired and languid; no benefit from sleep.) Now you see where Calc. comes in. *Generally better* in *warm* and *worse* in *cold* air. Great sensitiveness to open air.

Calc. has no marked chill and fever, but it may be curative in the chronic type, with its characteristic symptoms. Great sweating; morning sweat; the sweat is mostly upon the forehead and upon the feet. Clammy night-sweats, only on the legs. Feet are cold and damp, even during apyrexia. Calc. will follow Sulph. in many of these states.

TISSUES.—Cracking and crepitation in the joints, as if they were dry. The skin is rough and unhealthy; small wounds are disposed to suppurate; pale and flabby. Scurvy pimples. There are a great many eruptions upon the head and body where Calc. is indicated. Moist eruption behind the right ear. (Moist eruption exudes a glutinous fluid, honey-like—Graphites.) (If not glutinous and comes behind the ears and spreads to face—Sepia.) (If it spreads from the ears to the scalp—Hepar.) (Also, think of Dulc. in eruptions behind the ears.) (For ringworm always think of Sepia.) Warts.—The skin is rough, and inclined to chap. The eruptions of Calc. are aggravated in cold weather. The skin is bad in cold weather (Rhus. and Petroleum). In old scars that become inflamed and sore from every change of the weather, when the patient is in bad health.

---

THE readers of THE HOMŒOPATHIC PHYSICIAN will please excuse the delay in its appearance this month. It may not be generally known that the editor, Dr. Lee, is now in Europe and his duties have been performed by a deputy. The latter, having been somewhat prostrated by his professional labors, has sought rest and recuperation in a distant mountain region. This absence has caused consequent delay in the preparation of the journal. It is hoped to be able to issue the next number promptly on the first day of the month.

